# Advanced Practice Nursing Roles, Regulation, Education, and Practice: A Global Study

**DOI:** 10.5334/aogh.3698

**Published:** 2022-06-16

**Authors:** Kathy J. Wheeler, Minna Miller, Joyce Pulcini, Deborah Gray, Elissa Ladd, Mary Kay Rayens

**Affiliations:** 1 University of Kentucky College of Nursing, US; 2 British Columbia Children’s Hospital, CA; 3 George Washington University School of Nursing, US; 4 Old Dominion University School of Nursing, US; 5 MGH Institute of Health Professions, US

**Keywords:** advanced practice nurse, nurse practitioner, clinical nurse specialist, certified nurse midwife, nurse midwife, midwife, nurse in advanced practice

## Abstract

**Background and Objectives:**

Several subgroups of the International Council of Nurses Nurse Practitioner/Advanced Practice Nurse Network (ICN NP/APNN) have periodically analyzed APN (nurse practitioner and clinical nurse specialist) development around the world. The primary objective of this study was to describe the global status of APN practice regarding scope of practice, education, regulation, and practice climate. An additional objective was to look for gaps in these same areas of role development in order to recommend future initiatives.

**Methods:**

An online survey was developed by the research team, and included questions on APN practice roles, education, regulation/credentialing, and practice climate. The study was launched in August 2018 at the 10^th^ Annual ICN NP/APNN Conference in Rotterdam, Netherlands. Links to the survey were provided there and via multiple platforms over the next year.

**Results:**

Survey results from 325 respondents, representing 26 countries, were analyzed through descriptive techniques. Although progress was reported, particularly in education, results indicated the APN profession around the world continues to struggle over titling, title protection, regulation development, credentialing, and barriers to practice.

**Conclusions and Practice/Policy Relevance:**

APNs have the potential to help the world reach the Sustainable Development Goal of universal health coverage. Several recommendations are provided to help ensure APNs achieve these goals.

## Overview

The advanced practice nurse (APN) is an established healthcare provider delivering care throughout much of the world. In 2020, the International Council of Nurses defined the APN as:

a generalist or specialized nurse who has acquired, through additional graduate education (minimum of a master’s degree), the expert knowledge base, complex decision-making skills and clinical competencies for Advanced Nursing Practice, the characteristics of which are shaped by the context in which they are credentialed to practice (adapted from ICN, 2008). The two most commonly identified APN roles are Clinical Nurse Specialist (CNS) and Nurse Practitioner (NP) [1 (p6)].

Broadly speaking, NPs assess, diagnose, order and interpret laboratory tests, and prescribe medications for individual patients within a framework of collaboration with other medical providers and systems [2]. Though still involved in the direct provision of care to patients, CNSs tend to work more in healthcare administration and provide consultation and guidance to nursing staff and systems who manage complex patient care [3]. It is estimated about 40 countries currently have well-established APN roles [4]. Some of these countries have hundreds of thousands of APNs and others have more modest numbers.

Looking to the future, APNs may help counter the shortage and maldistribution of healthcare providers around the world. The World Health Organization predicts there will be a global deficit of 12.9 million physicians, nurses, and midwives by 2035 [5]. Physician roles and functions are fairly consistent throughout the world [6,7]. However, for APNs there are variations in the roles, titles, tasks, and regulatory, education and practice structures under which APNs provide care, country to country, and even jurisdiction to jurisdiction. Since 1999 several studies have attempted to document the evolution, expansion, and variation of the APN around the world (see Tables 1 and 2) [8,9,10,11,12]. These studies serve as snapshots in time of global role development and denote steady growth around the world and improving clarity of education, certification, and regulatory underpinnings.

**Table 1:** APN Role and Regulation Studies.


STUDY YEAR/S DATA COLLECTED/REPORTED	STUDY TITLE	INVITED PARTICIPANTS	KEY FINDINGS

**Research Subgroup of the ICN NP/APNN** [8] 1999–2000 collected 2001 reported	Survey Conducted at the ICN Centennial Conference in London	– ICN International Conference 1999 (London, England) – ICN NP/APNN Conference 2000 – ICN Nursing Association Members (120)	– 40 countries responded – education beyond that of a licensed or registered nurse in 33 countries, with education varied – only 26 countries reported education that led to a degree, diploma or certificate – education programs reported some kind of accreditation or approval process, though varied widely – some form of regulation reported in 13 of the countries

**Roodbol** [9] 2003–2004 collected 2004 reported	Survey Carried Out Prior to the 3^rd^ ICN INP/APNN Conference	– 3^rd^ ICN NP/APNN Conference (Gronigen, The Netherlands) – conference attendees	– over 60 countries reported either presence or interest in APN roles

**Pulcini, Jelic, Gul & Loke** [10] 2008 collected 2010 reported	An International Survey on Advanced Practice Nursing Education, Practice, and Regulation	– 174 key respondents and members of the ICN NP/APNN	– 32 responding countries – 13 titles identified for the NP/APN role – NP/APN education in 71% of 31 countries, with 50% recognizing master’s degree as prevalent credential – NP/APN role formally recognized in 23 countries, with 48% having licensure maintenance or renewal requirements – greatest support from domestic nursing organizations, individual nurses, and the government – greatest opposition from domestic physician organizations and individual physicians

**Heale & Buckley** [11] 2011 collected 2015 reported	An International Perspective of APN Regulation	– National Nursing Associations and global nursing health policy makers	– 30 responding countries with 26 reporting evidence of APN role – questions on regulation, education, scope of practice, opposition/barriers – significant variation in educational requirements, regulation, and scope of practice from country to country

**Maier & Aiken** [12] 2015 collected 2016 reported	Task Shifting from Physicians to Nurses in Primary Care in 39 Countries: A Cross-Country Comparative Study	– country experts suggested by group of international experts	– 39 responding countries with 27 reporting evidence of APN role via task shifting (England, Northern Ireland, Scotland, Wales all reporting separately) – 11 countries demonstrated significant task shifting, 16 countries demonstrated limited task shifting, 12 countries demonstrated no task shifting


**Table 2:** Country Responses Compared to Previous APN Studies.


PULCINI, JELIC, GUL & LOKE [10] NOTE*	HEALE & BUCKLEY [11] NOTE**	MAIER & AIKEN [12] NOTE***	CURRENT STUDY

	Angola		

Argentina			

Australia	Australia	Australia (1)	Australia

	Austria	Austria (3)	

	Bahrain		

		Belgium (2)	

	Bolivia		

Botswana	Botswana		Botswana

		Bulgaria (3)	

Canada	Canada	Canada (1)	Canada

			Chile

China			

		Croatia (2)	

		Cyprus (2)	

		Czech Republic (3)	

		Denmark (2)	

			Ecuador (role not established outside US agencies)

Ethiopia			

		Estonia (2)	

Figi			

Finland	Finland	Finland (1)	Finland

France	France	France (3)	France

		Germany (3)	Germany

			Ghana

	Greece	Greece (3)	

Grenada			

Hong Kong			

		Hungary (2)	Hungary

		Iceland (2)	

India			

	Iran		

Republic of Ireland	Republic of Ireland	Republic of Ireland (1)	Republic of Ireland

			Israel

Italy	Italy	Italy (2)	Italy

Jamaica			Jamaica

Japan			

			Kenya

		Latvia (2)	

		Lithuania (2)	

		Luxembourg (2)	

	Malaysia		

		Malta (2)	

	Mongolia		

Netherlands	Netherlands	Netherlands (1)	Netherlands

New Zealand	New Zealand	New Zealand (1)	New Zealand

Nigeria			

		Norway (3)	

Oman			

Pakistan			

	Poland	Poland (3)	

Portugal		Portugal (2)	Portugal

		Romania (3)	

Saudi Arabia/KSA			

	Sierra Leone		

Singapore			Singapore

		Slovakia (3)	

		Slovenia (2)	

	Spain	Spain (2)	Spain

South Africa			

South Korea			

		Sweden (2)	

Switzerland	Switzerland		

Taiwan	Taiwan		

Tanzania			Tanzania

Thailand	Thailand		

	Togo		

		Turkey (3)	

		Switzerland (3)	

United Kingdom	United Kingdom	United Kingdom (1) England Northern Ireland Scotland Wales	United Kingdom England Northern Ireland Scotland Wales

United States	United States	United States (1)	United States


* Unclear if participation in survey demonstrated presence of APN role. ** Role present in all countries, though significant variation in regulation and education. *** Level of task shifting as follows: 1 = significant task shifting, 2 = limited task shifting, 3 = no task shifting.

Although titles, roles, and duties vary around the world, *advanced practice nurse* is a commonly accepted umbrella term representing four generally established advanced roles—the two described above, NP and CNS, as well as nurse anesthetist and nurse midwife. And while APN is a broadly accepted representative term, most countries and jurisdictions use other terms to refer to nurses who practice in an advanced role. For instance, the title adopted in the United States (US) is Advanced Practice Registered Nurse (APRN), specifically developed by the Consensus Model for APRN Regulation in 2008 [13]. Aside from codifying the titles of the four disciplines representing APRNs--Certified Nurse Practitioner (CNP), Clinical Nurse Specialist (CNS), Certified Nurse Midwife (CNM), and Certified Registered Nurse Anesthetist (CRNA)—the Consensus Model sought to ensure consistency in licensure, accreditation, certification, and education, facilitating regulation of APRNs throughout the US. The Consensus Model, which was adopted in the US in 2008, is a rather recent development relative to the observation that varied APN roles have existed in some form for over a hundred years [14,15]. The first global definition occurred in 2002, when the ICN defined an NP and APN, and the master’s degree was only a recommendation [16]. The more recent ICN definition of the APN, provided above, set the master’s degree as the minimal education requirement and emphasized an advanced level of decision-making and responsibility. However, it did not include definitions for APNs who deliver anesthesia or who participate in childbirth.

To describe the global status of APN practice regarding scope of practice, education, regulation, and practice, the Health Policy Subgroup of the International Council of Nurses Nurse Practitioner/Advanced Practice Nurse Network (ICN NP/APNN) recently completed this global study. An additional objective was to look for gaps in these same areas of role development in order to recommend future initiatives.

## Methods

### Survey Development

An online survey was developed by the research team, drawn largely from the 2010 Pulcini, Jelic, Gul, and Loke survey [10] as well as regulatory questions from the 2015 Heale and Buckley survey [11], once adaptation permission was granted. Questions were refined, with several areas added or developed, most notably the modification of questions on education, professional issues, clinical skills, credentialing, and certification. The survey categorized questions according to practice roles, education, regulation/certification, and practice climate. Because of the complexity of APN titling and practice issues, respondents were given the opportunity to answer multiple questions with open-ended responses in addition to multiple choice options. To clarify distinctions of education and credentialing, definitions for *title protection, certificate, certification*, and *recertification* were provided (see Table 3).

**Table 3:** Education and Credentialing Definitions Provided in Survey for Items Needing Clarification.


TERM	DEFINITION AS PROVIDED IN SURVEY

**Title protection**	Title protection, as adapted from the American Nurses Association definition, refers to the restricted use of the title to only those individuals who have fulfilled the requirements for the licensure/recognition in each jurisdiction’s legislation/regulations/rules so as to protect the public against unethical, unscrupulous, and incompetent practitioners.

**Certificate/certification**	To clarify the difference between the meaning of “certificate” and the meaning of “certification” the following definitions are provided by the American Accreditation Board for Nursing Specialties: Certificate program refers to “an educational program that awards a certificate after completing the program.” Certification refers to “an earned credential that demonstrates the holder’s knowledge, skills and experience. It is awarded by a third party.” Generally the third party is non-governmental but, in some situations, could be a governmental agency.

**Certification/recertification**	Certification, as defined by the American Accreditation Board of Nursing Specialties, refers to “an earned credential that demonstrates the holder’s knowledge, skills and experience. It is awarded by a third party…” Generally the third party is non-governmental but, in some situations, could be a governmental entity. Conditions for certification usually involve experience, education and an exam. Conditions for recertification usually involve experience and continuing education, but may involve another exam. Certification is a formal recognition of an individual’s education, skills and practice AS OPPOSED to licensure/registration/endorsements, which is an individual’s formal authorization to practice.


### Procedure

Once the research team obtained institutional review board (IRB) approval from the Office of Research Integrity of the University of Kentucky and the survey was approved by the Core Steering Group of the ICN NP/APNN, the study was launched in August 2018 at the 10^th^ Annual ICN NP/APNN Conference in Rotterdam, Netherlands. Links to the survey were provided there and via ICN social media platforms. When initial data analysis showed response gaps from several continents due to institutional firewalls, the IRB approval was amended to allow document surveys to be anonymously submitted with deadline extended to September 2019.

### Design and Sample

A convenience sample approach was used because of the difficulty accessing all eligible participants or countries worldwide. Participants completed the survey once in this cross-sectional assessment. Study participants were required to be APNs, APN educators, APN administrators, and/or APN researchers; be fluent in reading/writing English; and have access to a computer with an Internet connection. Completion of the survey established participant consent. Survey responses from 325 respondents, representing 26 countries, were analyzed. However, the study data were summarized as being from 23 countries, with data from England, Northern Ireland, Scotland, and Wales, combined as a single location category (i.e., United Kingdom [UK]).

### Analysis

Each survey was analyzed for sufficiency of response. Participants from all represented countries answered both multiple choice and open-ended questions. We received 482 surveys in total, but 157 of them were not able to be retained due to widespread missing values; the effective sample size was 325, reflecting 67% of the total surveys received. Descriptive statistics, including frequencies and percentages, were used to analyze and describe the sample data. SAS, v. 9.4 was used for the quantitative analysis.

## Findings

### Demographics

Responses came from countries in all the major regions of the world, specifically Africa (*n* = 4), Asia (*n* = 2), Europe (*n* = 10), North America (*n* = 3), South America (*n* = 2) and Oceania (*n* = 2), as presented in Table 4.

**Table 4:** Country Respondents.


COUNTRY	N	PERCENT

**Australia**	5	1.54

**Botswana**	2	0.62

**Canada**	85	26.15

**Chile**	3	0.92

**Ecuador (role not established outside US agencies)**	1	0.31

**Finland**	4	1.23

**France**	4	1.23

**Germany**	3	0.92

**Ghana**	3	0.92

**Hungary**	1	0.31

**Israel**	1	0.31

**Italy**	1	0.31

**Jamaica**	1	0.31

**Kenya**	2	0.62

**Netherlands**	39	12.00

**New Zealand**	4	1.23

**Portugal**	3	0.92

**Republic of Ireland**	5	1.54

**Singapore**	3	0.92

**Spain**	10	3.08

**Tanzania**	1	0.31

**United Kingdom** **England** **Northern Ireland** **Scotland** **Wales**	41	12.62

**United States**	103	31.69


Demographic totals and percentages are presented in Table 5.

**Table 5:** Demographics of Survey Respondents.


PRACTICING NURSES	*N*	PERCENT*

Registered/Generalist Nurse	71	21.82

Hospitalist/Acute Care NP/APN	48	14.77

Specialty care specific to disease or illness NP/APN	50	15.38

Specialty care specific to an age group or population NP/APN	32	9.85

Family NP/APN	121	37.23

Geriatric/Gerontologic NP/APN	18	5.54

Paediatric NP/APN	17	5.23

Adult NP/APN	45	13.85

Adult Gerontologic NP/APN	7	2.15

Women’s Health NP/APN	12	3.69

Midwife	2	0.62

Community Health NP/APN	21	6.46

Mental Health NP/APN	17	5.23

CNS	22	6.77

Other	47	14.46

**EDUCATORS**	***N***	**PERCENT**

Registered/Generalist Nurse	18	11.69

NP/APN	65	42.21

Both of above	59	38.31

Other	12	7.79

**ADMINISTRATORS**	***N***	**PERCENT**

Nursing personnel	22	48.89

Non-nursing personnel	0	0

Both of above	23	51.11

**RESEARCHERS**	***N***	**PERCENT**

Nursing related	56	52.83

Non-nursing related	6	5.66

Both of above	44	41.51

**OTHER ROLES**	***N***	**PERCENT**

	73	


* Percentages do not add to 100% because respondents could select more than one answer.

Eligible participants could identify themselves as practicing nurses, educators, administrators, and/or researchers. Because nurses (and those associated with nurses) function in many roles, respondents were asked to check all that applied in each category or provide additional roles if an option was not listed. For this reason, the cumulative percentage across all roles exceeds 100%.

Of respondents who reported practicing as nurses, 37% (*n* = 121) identified as a Family NP/APN, 22% (*n* = 71) as a registered/generalist nurse, 15% (*n* = 50) as a NP/APN specialist devoted to a specific disease, 15% (*n* = 48) as a Hospitalist/Acute Care NP/APN, 14% (*n* = 45) as an Adult NP, 10% (*n* = 32) as a NP/APN specialist devoted to a specific age or population, 7% (*n* = 22) as a Clinical Nurse Specialist, 6% (*n* = 18) as a Geriatric/Gerontologic NP/APN, 6% (*n* = 21) as a Community Health NP/APN, 5% (*n* = 17) as a Mental Health NP/APN, 5% (*n* = 17) as a Paediatric NP/APN, 4% (*n* = 12) as a Women’s Health NP/APN, 2% (*n* = 7) as an Adult/Gerontologic NP/APN, and 1% (*n* = 2) as a Midwife. The 14% (*n* = 47) of practicing nurses who reported roles outside those offered in the survey listed roles such as neonatal nurse practitioner or nurse anesthetist.

Of respondents who identified as educators, 12% (*n* = 18) reported they educated registered/generalist nurses only, 42% (*n* = 65) educated APNs only, and 38% (*n* = 59) reported they educated both. An additional 8% (*n* = 12) reported educating students other than registered/generalist nurses or APNs. Those who identified as administrators were almost equally split, with 49% (*n* = 22) reporting oversight of nursing personnel and 51% (*n* = 23) reporting oversight of both nursing and non-nursing personnel. Over half of the researchers reported they were involved exclusively in nursing research (53% (*n* = 56), 6% (*n* = 6) in non-nursing research, and 42% (*n* = 44) in both.

### Practice Role

Practice questions centered on titling and types of APN roles, presence/absence of title protection, professional issues, and clinical skills (see Appendix A). Most countries with some sort of APN practice reported more than one advanced role. Though most used the titles NP, CNS, or midwife, other titles were listed, such as APN, nurse in advanced practice, expert nurse, nurse specialist, and others. In some countries the term CNS (or a similar title) referred to nurses who function more as NPs, or vice versa (i.e., providers titled NPs but who functioned more as CNSs). Some countries reported midwives were commonly educated at the registered/generalist level or as a non-nurse, while other countries reported educating midwives at a post registered/generalist nurse level. Title protection was reported in Australia, Botswana, Canada, France, Hungary, Israel, Jamaica, the Netherlands, New Zealand, Portugal, Republic of Ireland, Singapore, and the US. Title protection was not reported in Chile, Finland, Germany, Ghana, Italy, Kenya, Spain, Tanzania, or the UK.

Respondents chose from 15 APN work-place position options, such as doctor’s office, hospital-based clinic, hospital, faculty, and the like. Respondents could report all that applied as well and were able to list any unnamed workplace settings in an open-ended question. Australia, Botswana, Canada, Finland, the Netherlands, New Zealand, Spain, the UK, and the US responded affirmatively to all site options. Portugal reported all site options except occupational/workplace health, while the Republic of Ireland reported all site options except school health and occupational or workplace health. Singapore reported a little over half the work site options, while the remaining countries reported fewer than half of the work site options. Israel reported only specialty practice sites and Hungary reported that the role was too recently instituted to provide any details. Ecuador reported the role did not exist outside US government agencies, so will only be reported in the tables but not included in discussions or subsequent calculations. Other questions pertained to 21 clinical skills (from skin lesion removal to suturing to X-ray interpretation) and 12 professional issues (from carrying their own caseload of clients/patients to ability to prescribe to reimbursement (see Appendix A).

### Education

Education questions pertained to presence/absence of programs, number of programs, level of education, types of specialties or APNs, program details, and student requirement details (see Table 6).

**Table 6:** Education.


COUNTRY	*N*	FORMAL EDUCATION, NO. OF PROGRAMS	EDUCATION CREDENTIAL	TYPES OF EDUCATION FOR NPS/APNS*	PROGRAM DETAILS	STUDENT REQUIREMENT DETAILS

**Australia**	5	Yes, >10	Doctorate, master’s	a-m	2 yr. full time/3 yr. part time 300 dedicated clinical, 5000 hours before endorsement as NP Funding by government and/or student	Registration as RN/Generalist Nurse Academic degree Minimum 2 yrs. as RN in specified clinical field and2 yrs. of current advanced nursing practice in same clinicalfield

**Botswana**	2	Yes, <10	Master’s, baccalaureate, advanced diploma	a, d, f, g, j, k, l, m	2 yr. full time 500 clinical hours Funding by government and/or student	Registrationas a RN/Generalist Nurse Academic degree Minimum 2 yearsas RN/Generalist Nurse

**Canada**	85	Yes, >10	Doctorate, master’s, baccalaureate, certificate, advanced diploma	a-m, n (anaesthesia/anesthetist, neonatal, primary care)	Programlength varies according to school and degree Clinical hours varyaccording to school and degree Funding by government, private funding, and/or student	Registration as a RN/Generalist Nurse Academicdegree Minimum of 2 years as RN/Generalist Nurse

**Chile**	3	Yes, <5	Master’s	m, n (degree generic, considered = to MSN)	2 yr. full time 500–800 clinical hours Funding by student	Registration as a RN/Generalist Nurse Academic degree Minimum of 3 years as RN/Generalist Nurse

**Ecuador (role not established outside US agencies)**	1	No	N/A	N/A	N/A	N/A

**Finland**	4	Yes, <5	Master’s, certificate, advanced diploma	a, b, g, i, l, m	Program length varies according to school and degree Clinical hours vary according to school and degree Funding by government or employer	Registration as aRN/Generalist Nurse Academic degree Minimum of 3 years asRN/Generalist Nurse, more if they will be a prescriber

**France**	4	Yes, <5	Master’s, advanced diploma	b, e, k, l, n (oncology, nephrology)	Program length varies according to school and degree No response to clinical hours question Funding by government	Registration as a RN/Generalist Nurse Academic degree Minimumof 3–5 years as RN/Generalist Nurse, depending on schooland degree

**Germany**	3	Yes, <5	Doctorate, master’s, baccalaureate, no credential is granted	b, c, e, g, h, k, l, m	2–3 yr., depending on school and degree No response toclinical hours question Funding by government and/or student	Registration as a RN/Generalist Nurse Academic degree Minimum of 1–2 years as RN/Generalist Nurse, depending on school and degree

**Ghana**	3	Yes, <5	Baccalaureate, advanced diploma	a, f, g, l, m, n (general nurse practitioner)	2–3 yr. full time Noresponse to clinical hours question Funding by student	Registration as a RN/Generalist Nurse Academic degree Minimum of 3 years as RN/Generalist Nurse

**Hungary**	1	Yes, <5	Master’s	a, c, e, k, n (anesthesiology, perioperative)	18 mo. full time 1490 clinical hours No response to source of funding	Registration as a RN/Generalist Nurse Academic degree Minimum of 3 years as RN/Generalist Nurse

**Israel**	1	Yes, <5	Certificate	No response	No response	No response

**Italy**	1	Yes, <5	Doctorate, master’s	d, e, g,	2 yr. full time 500 clinical hours Funding by student	Registration as a RN/Generalist Nurse Academic degree

**Jamaica**	1	Yes, <5	Master’s	d, f, g, j, l, m	2–3 yr. full time No response to clinical hours question Funding by student	Registration as a RN/GeneralistNurse Academic degree Minimum of 2 years as RN/GeneralistNurse

**Kenya**	2	Yes, <10	Master’s	b, j, k	No response regarding program length, clinical hours or funding	Registration as aRN/Generalist Nurse Academic degree Minimum of 2 years asRN/Generalist Nurse

**Netherlands**	39	Yes, >10	Master’s, baccalaureate, certificate, advanced diploma	a-m, n (other: five APN types-acute, preventive, intensive, chronic and mental health. Soon only general healthcare and mental healthcare. GYN skills transferred to nurse specialists)	2 yr., though one program 3 yr. Unclear response to clinical hours question Funding by government, private funding, student and/or other	Registration as a RN/Generalist Nurse Academic degree Minimum of 2 years as RN, Generalist Nurse

**New Zealand**	4	Yes, <10	Doctorate, master’s, baccalaureate, certificate, advanced diploma	All except midwife. Midwives are not considered APNs.	2–5 yr. full time 300–500 clinicalhours Funding by government, private funding, and/or student	Registration as a RN/Generalist Nurse Academic degree Minimum of 4 years as RN/Generalist Nurse

**Portugal**	3	Yes (for clinical specialist, specialist nurse), <10	Master’s, certificate	a, d, f, i, j, k, l, m, n (rehabilitation)	18 mo. No response to clinical hours question No response to funding question	Registration as a RN/Generalist Nurse Academic degree

**Republic of Ireland**	5	Yes, <10	Master’s, advanced diploma	a-m	2 yr. FT 500 clinical hours + 100 hours prescribing question Funding bygovernment or other	Registration as a RN/Generalist Nurse Academic degree Minimum of 3 years as RN/Generalist Nurse

**Singapore**	3	Yes, <5	Master’s	a, b, c, d, e, f, g, h, i, k, l	2 yr. plus 1 yr. internship, under review to reduce to 18 mo. 800 clinical hours, increasing to 1200 in 2020, 12 mo. internship Funding bygovernment and/or other	Registration as a RN/Generalist Nurse Academic degree Minimum of 5 years as RN/Generalist Nurse

**Spain**	10	Yes, <5	Doctorate, master’s, certificate, advanced diploma	a, b, d, e, f, h, i, j, k, l, m, n (emergency nurse anesthetist)	2 yr. full time No response to clinical hours question Funding by government and/or student	Registration as a RN/Generalist Nurse Academic degree

**Tanzania**	1	Yes, <5	Doctorate, master’s	j, l,	2 yr. full time Noresponse to clinical hours question Funding by government and/orstudent	Registration as a RN/Generalist Nurse Academic degree Minimum of 1 years as RN/Generalist Nurse, though 3 preferred

**United Kingdom**	41	Yes, >20	Doctorate, master’s, baccalaureate, certificate, advanced diploma, no credential is granted, other (unspecified)	All & n (neonatal)	2–3 yr. full time 500–1000 clinical hours according to school and degree Funding by government, private funding, student and/or other	Registrationas a RN/Generalist Nurse Academic degree Minimum of 2–7years as RN/Generalist Nurse

**United States**	103	Yes, >20	Doctorate, master’s, baccalaureate, certificate, advanced diploma	a-m, n (nurse anesthetist)	18 mo. to 4 years depending on school and degree 500–1200 clinical hours according to school and degree Funding by government, private funding, student and/or other	Registration as aRN/Generalist Nurse Academic degree Number of years of experienceas RN/Generalist Nurse varies according to school and program


Low response countries in shaded gray. * a = Hospitalist/acute care NP/APN, b = specialty care specific to disease or illness NP/APN, c = specialty care specific to an age group or population NP/APN, d = family NP/APN, e = geriatric/gerontologic NP/APN, f = paediatric NP/APN, g = adult NP/APN, h = adult gerontologic NP/APN, i = women’s health NP/APN, j = midwife, k = community health NP/APN, l = mental health NP/APN, m = clinical nurse specialist, n = other.

All the countries reported having formal education programs for APNs. Only Australia, Canada, the Netherlands, the UK, and the US reported more than ten such programs in their country, with the remainder reporting fewer than this. Most of the countries offered multiple education paths for those wanting to practice as APNs. All the countries except Ghana and Israel listed the master’s degree as the education credential available to APN graduates, with Ghana offering the baccalaureate and advanced diploma, and Israel offering a certificate. Canada, New Zealand, and the US reported all five levels of education (doctorate, master’s, baccalaureate, certificate, advanced diploma), but Canada and the US specified those who earned the lower credentials had done so before the master’s had been required and had been grandfathered into practice. The UK reported all five levels of education but also reported some programs educated APNs but granted no credential. Germany reported offering doctorate, master’s, and baccalaureate degrees as well as programs of APN education where no credential was granted.

Australia, Canada, the Netherlands, the UK, and the US reported education for all advanced roles, though New Zealand participants specified midwifery was not considered an advanced role. Other than Israel, the remaining countries reported a variety of roles for which there were APN programs, including some listing disease-specific programs (e.g., an oncology APN track).

Reports of program length varied from 18 months to five years according to program type and degree, with most reporting programs that require two to three years of full-time schooling. Several programs reported a minimum of 500 clinical hours, though some required considerably more (e.g., 800; 1000; 1200; or 1490), additional internships (e.g., one year long; another as long as 5000 hours), and one specified clinical hours specifically devoted to pharmacology (in addition to other hours required).

All the countries with APN programs reported requiring students to be registered/generalist nurses with academic degrees before entering the program. With the exception of the US, nearly all the programs required registered/generalist nurses to have a minimum of two years of experience as a nurse, with some requiring as many as seven years.

### Regulation and Credentialing

Regulation and credentialing questions pertained to presence/absence of recognition, regulation level, requirements to practice, requirements to renew, and certification (see Table 7).

**Table 7:** Regulation, Credentialing, and Certification.


COUNTRY	*N*	FORMAL RECOGNITION LEVEL	REGULATION LEVEL	REQUIREMENTS TO PRACTICE	RN/POST RN PRACTICE LEVEL	SPECIFIC REQUIREMENTS TO RENEW	REGULATORY MODEL

**Australia**	5	Government, hospital/health care agency, professional organizations	Federal, jurisdictional	Academic degree, approved education program, registration/licensure/endorsement by government agency	Post RN	Continuing education, portfolio, practice	Registration/licensure/endorsement at national level Tasmania has similar process as above

**Botswana**	2	Government, hospital/health care agency, professional organizations	Federal	Academic degree, approved education program, registration/licensure/endorsement by government agency	Post RN	Continuing education, portfolio, practice	Registration/licensure/endorsement at national level

**Canada**	85	Government, hospital/health care agency, professional organizations	Federal, jurisdictional	Academic degree, approved education program, registration/licensure/endorsement by government agency, certification by a non-governmental agency	Post RN*	Continuing education, portfolio, practice	Registration/licensure/endorsement at jurisdictional level Certification exam by independent agency

**Chile**	3	Role recognized but no formal government regulation, professional organizations in existence	N/A	Approved education program	RN/Post RN	N/A	Registration/licensure/endorsement at agency level

**Ecuador (role not established outside US agencies)**	1	N/A	N/A	N/A	N/A	N/A	N/A

**Finland**	4	Role recognized but no formal government regulation	N/A	Academic degree, approved education program	RN/Post RN	N/A	Registration/licensure/endorsement at agency level

**France**	4	Government, hospital/health care agency, professional organizations	Federal	Academic degree, approved education program	RN/Post RN	Portfolio	Registration/licensure/endorsement at national level

**Germany**	3	Role recognized but no formal government regulation, professional organizations in existence	N/A	Academic degree, approved education program	Post RN	N/A	Registration/licensure/endorsement at agency level

**Ghana**	3	Government, professional organizations	Jurisdictional	Academic degree, approved education program, registration/licensure/endorsement by government agency	RN/Post Rn	Continuing education, practice	Registration/licensure/endorsement at jurisdictional level

**Hungary**	1	Government, professional organizations	Federal	Academic degree, approved education program, registration/licensure/endorsement by government agency	Post RN	Continuing education, portfolio	Registration/licensure/endorsement at national level

**Israel**	1	Government, professional organizations	Federal	Academic degree, approved education program, registration/licensure/endorsement by a governmental agency, certification exam by a governmental agency	Post RN	N/A	Registration/licensure/endorsement at national level Certification exam

**Italy**	1	Role recognized but no formal government regulation	N/A	Academic degree	Post RN	N/A	Registration/licensure/endorsement at agency level

**Jamaica**	1	Role recognized but no formal government regulation	N/A	Approved education program	Post RN	Continuing education, practice	Registration/licensure/endorsement at agency level

**Kenya**	2	Role recognized but no formal government regulation, professional organizations in existence	N/A	Approved education program	RN	N/A	Registration/licensure/endorsement at agency level

**Netherlands**	39	Government, hospital or health care agency, professional organization	Federal	Academic degree, approved education program, registration/licensure/endorsement by a governmental agency	Post RN**	Continuing education, portfolio, practice	Registration/licensure/endorsement atnational level

**New Zealand**	4	Government, hospital or health care agency, professional organization	Federal, jurisdictional	Academic degree, approved education program, registration/licensure/endorsement by a governmental agency	RN/Post RN	Continuing education, portfolio, practice	Registration/licensure/endorsement at national level

**Portugal**	3	Government, hospital or health care agency, professional organization	Federal	Academic degree, approved education program, registration/licensure/endorsement by a governmental agency	RN/Post RN	N/A	Registration/licensure/endorsement at national level

**Republic of Ireland**	5	Government, hospital or health care agency, professional organization	Federal, jurisdictional	Academic degree, approved education program, registration/licensure/endorsement by a governmental agency	RN/Post RN	Continuing education, portfolio, practice	Registration/licensure/endorsement at national level

**Singapore**	3	Government, hospital or health care agency, professional organization	Federal	Academic degree, approved education program, registration/licensure/endorsement by a governmental agency	Post RN	Continuing education, practice	Registration/licensure/endorsement at national level

**Spain**	10	Role recognized but no formal government regulation	N/A	Academic degree, approved education program, registration/licensure/endorsement by a governmental agency, registration/licensure/endorsement by a non-governmental agency, sponsorship by a clinical agency	RN/Post RN	N/A	Registration/licensure/endorsement at agency level

**Tanzania**	1	Role recognized but no formal government regulation	N/A	Academic degree	Post RN	Continuing education	Registration/licensure/endorsement at agency level

**United Kingdom**	41	In infancy at government, hospital or health care agency, professional organization***	N/A	Academic degree, approved education program, registration/licensure/endorsement by a non-governmental agency***	RN/Post RN	Note***	Registration/licensure/endorsement at agencylevel Note***

**United States**	103	Government, hospital or health care agency, professional organization	Federal, jurisdictional	Academic degree, approved education program, registration/licensure/endorsement by government agency, certification examination by a non-governmental agency	Post RN****	Continuing education, portfolio, practice	Registration/licensure/endorsement at jurisdictional level Certification exam


Low response in shaded gray. *Some NPs were grandfathered to role without master’s degree but met other education/exam requirements. They tend to practice in remote areas. **The APN, entitled Nurse Specialist, is issued a credential of qualification upon graduation from a professional program. Over the next five years the APN must demonstrate a specified level of practice, continuing education and a number of other professional activities. If the APN cannot provide proof of these activities the APN is struck from the Registry, losing the Nurse Specialist title. The title can be reestablished by entering an individual educational program with a licensed MANP program. At the conclusion of the program the credential is re-earned and the individual can register as Nurse Specialist with the Registration Commission. ***Government starting to place requirements on advanced nursing practice but in infancy. RCN offers a credentialing process, but it is not compulsory. Some groups campaigning for formal entry on the NHS. The Royal College of Emergency Medicine and the Faculty of Intensive Care Medicine both offer a credentialing process and associate membership. ****Some NPs were grandfathered to role before master’s was required. Now rare since most are retired or close to retirement.

Nearly half (45%, *n* = 10) reported formal recognition by the government, hospital/health care agency, and/or professional organizations in their countries and some (14%, *n* = 3) reported formal recognition by the government and professional organizations only. The remaining (40%, *n* = 9) reported the role was recognized though there were no formal regulations at any governmental level. We assumed that the APNs working in these countries were credentialed by the local agencies employing them.

For those who reported regulation by a governmental agency, most regulation was reported at the federal level. However, Australia, Canada, New Zealand, the Republic of Ireland, and the US reported jurisdictional level regulation as well. Only Ghana reported regulation solely at the jurisdictional level.

All the countries reported requiring an academic degree and/or approved education program in order to enter practice as an APN, though some reported grandfathering had occurred in the past for experienced APNs who would not be able to meet current education standards. Over half the countries (64%, *n* = 14) required registration, licensure, or endorsement at some governmental level to practice. Of the remaining countries (36%, *n* = 8), registration, licensure or endorsement to practice was listed at agency level authorization. Canada, Israel, and the US required passage of a certification exam in order to practice. Requirements to continue to practice mostly involved maintenance of practice, earning continuing education credits, or meeting portfolio requirements on some interval basis.

### Practice Climate

Practice climate questions pertained to factors that facilitated or hindered APN role development and level of policy making and professional group organization (see Appendix B).

Nearly all respondents (73%, *n* = 16) reported the basis for development of the APN role was due to a need for providers in rural or underserved areas, with several (23%, *n* = 5) reporting very specific physician shortage issues in neonatal care or psychiatry, or policy changes that limited work hours of residents or junior doctors. Nearly all (77%, *n* = 17) reported consumer demand led to APN role development. One respondent reported that extensive dialogue had gone on about providing the right (high quality) care in the most (cost) efficient way, as well as patient needs moving from “illness and cure” to “health and behavior”.

Responses about who specifically advocated for or opposed the role were mixed. Advocacy options included the following: government; international organizations; individual nurses; individual physicians; consumers; insurance companies; universities; media; and/or in-country nursing, physician, nongovernmental/nonprofit institutions or private institutions. Two countries reporting affirmatively for all, but the remaining cited a mixture of responses. Over half of the countries (68%, *n* = 15) reported that government and nursing organizations within country were the prime advocates for the APN role. Over a third (37%, *n* = 8) reported physician organizations within country advocated for the role as well. However, regarding role opposition, physician organizations were reported the most (73%, *n* = 16), followed by individual physicians (68%, *n* = 15), individual nurses (55%, *n* = 12), and governments (45%, *n* = 10).

The majority of the countries (73%, *n* = 16) reported that policy making for APNs occurred at both a national and local level, though the respondent from Jamaica reported it occurred at the local level and the respondent from Italy reported it occurred at the national level. The development of APN organizations was reported mostly (73%, *n* = 16) at both the national and local level but the respondent from Chile reported it only at the national level, while France and Ghana reported it only at the local level. Hungary reported no evidence of either.

## Discussion

Larson states characteristics of a profession include a “professional association, cognitive base, institutionalized training, licensing, work autonomy, colleague control” [17 (p208)]. This research found significant evidence APNs possess the characteristics of a profession in many places around the world. But ongoing variations and gaps continue, and these gaps certainly have the potential to impact the profession as well as the care APNs provide and the ability to expand health care to those in need.

### Distribution of APNs

Understanding the number, distribution, and types of providers present in the world is extremely complicated. It is even more complex for APNs, not only because of inherent problems of workforce data collection but also due to issues of categorization unique to nursing and APNs. The World Health Organization collects data on healthcare professionals throughout the world but admits the quality and completeness of the data is a concern [18]. Categories of collection include medical doctors, nursing and midwifery personnel, dentistry, and a few others. There is no separate category for APNs, their presence being counted among the nurses and midwives. This same limitation of data collection on APNs persists throughout many of the countries and jurisdictions of the world. Nonetheless, it is known NPs and CNSs are established in the Americas (US [19], Canada [19], Jamaica [20], Belize [20], Brazil [20], Chile [20], and Columbia [20], among others), much of Europe (Austria [21], Belgium [22], Czech Republic [22], Finland [22], France [22], Germany [23], Republic of Ireland [22], the Netherlands [19], Poland [22], Switzerland [23], the UK [22], among others), Australia [22], New Zealand [19], a few countries in Africa (Botswana [19], Ghana [24], Eswatini [25], Kenya [19], Namibia [19], and Zimbabwe [19], among others), and a few countries in Asia (Israel [23], Japan [22], Oman [19], Singapore [19], and Taiwan [26], among others). This aligns well with the responses obtained not only from this study but also those cited in Table 2, and may point to areas of the world that could potentially benefit from NP and CNS role introduction.

### Titling, Role, and Practice

Inconsistencies in titling, role, and practice continue to affect the profession. If individuals do not need to work outside the country or jurisdiction, the variations are not inherently limiting. However, healthcare needs are not always geographically bound, nor are the needs of professionals who sometimes must move for personal or professional reasons. Numerous authorities indicate this lack of standardization limits the ability of APNs to meet unmet healthcare needs, collaborate across borders, partake in scholarly exchanges with a common language, or participate in dependable and consistent research on the profession or the outcomes of care [27,28,29].

Title protection is also a critical professional issue, regardless of geographic mobility. Since title protection is the limited use of a title unless the title holder meets regulatory requirements [30] the finding that nearly half of the countries reported no title protection causes concern. The American Nurses Association states title protection protects the public from “unethical, unscrupulous, and incompetent practitioners” [31 (para 1)]. It also protects the practitioner from unfair competition from someone who does not meet education or regulatory standards. Additionally, having a defined and protected title provides regulators and the public with a common and understood frame of reference from which to create sound regulations and measure, monitor, and discipline the profession.

### Education

That nearly all the countries reported the master’s degree as the primary form of education for APNs is evidence the 2002 ICN recommendation of a master’s degree for advanced practice has had an impact. It is noteworthy these programs also required similar entry criteria, as well as similar clinical requirements and program length. The greatest variation was found in program and role offerings available by schools or within countries, a likely variation dictated by local need or knowledge of available roles. However, while this local determination might meet current local needs it could also limit geographic flexibility or the ability to attend to future, evolving needs. And local determination could be very restrictive to the nurse who wants to do something outside of what is locally available but has limited resources to seek education elsewhere. Unfortunately, this study did not look at educational curricula or program accreditation.

### Regulation and Credentialing

The most difficult professional area to understand and describe for the status of APNs around the world is regulation and credentialing. In 1997, as a concept fundamental to regulation, the ICN defined credentialing as:

processes used to designate that an individual, programme, institution or product have met established standards set by an agent (governmental or non-governmental) recognised as qualified to carry out this task. The standards may be minimal and mandatory or above the minimum and voluntary. Licensure, registration, accreditation, approval, certification, recognition or endorsement may be used to describe different credentialing processes…Credentials may be periodically renewed as a means of assuring continued quality and they may be withdrawn when standards of competence or behavior are no longer met [32 (p44)].

Because this terminology is complicated, with terms used interchangeably, APNs may not be able to fully describe the level of credentialing involved in the ability to practice or teach, which was reflected in this study. Considerable cross referencing had to be done to understand the regulatory models of the countries represented. Clearly, many APNs work and practice without the benefit of regulation at any governmental level. What remains for these APNs is agency recognition, a level of credentialing that may cause concern and confusion for the public.

Certification is another term used in a variety of ways as a credential descriptor. For the purposes of this study, professional certification was defined as:

the voluntary process by which a(n)…entity grants a time-limited recognition and use of a credential to an individual after verifying that he or she has met predetermined and standardized criteria. It is the vehicle that a profession or occupation uses to differentiate among its members, using standards sometimes developed through a consensus-driven process, based on existing legal and psychometric requirements [33 (p5)].

While certification can refer to a level of recognition based on curriculum, this study looked specifically for competency-based certification (exam-based) as well as portfolio- based competency. Though considered the best measure of competency, few countries relied on this level of certification for credentialing.

Table 8 provides a comparison of positive and negative factors related to level of regulation and certification.

**Table 8:** Regulatory Models: Advantages and Disadvantages.


		REGULATORY MODEL	POSITIVE FACTORS	NEGATIVE FACTORS

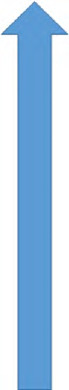	**Competency Based Credential**	**Centralized/decentralized regulation + Certification by Exam (including maintenance)**	Favored by most authorities	Requires significant resources todevelop/deliver exams

		**Centralized/decentralized regulation + Certification by Portfolio (including maintenance)**	Favored by most authorities	Requires significant resources to reviewportfolios

	**Curriculum Based Credential**	**National (centralized) regulation**	Can advance role if kept up-to-date [34]	Can negatively impact role if not kept up-to-date [34]

		**Jurisdictional (decentralized) regulation**	Meets local needs	Leads to inconsistencies in practice and role clarity [34] Requiressignificant time and resources to change [34]

		**Agency regulation**	Meets agencyneeds	Leads to greater inconsistencies in practice and role clarity [34] Results in limited data for the profession [34]

	**Limited Competency Credential**	**Agency regulation**	May meet care needs at some level	Increasespotential for incompetent providers Leads to inconsistencies in practice androle clarity Requires coordinated effort to move to higher levelcredential Results in limited data for the profession


Clearly, regulatory models matter significantly to the practice of APNs and the potential for expansion of care they could provide. At the same time, recognition must be given to the effort that has already gone into developing the role and which will be required for future regulatory model expansion.

### Practice Climate

Despite nearly universal reporting of a need for providers in rural or underserved areas, as well as consumer demand, practice climate continues to negatively impact the profession, even where the APN role has functioned for decades. A 2009 study by the Organization for Economic Cooperation and Development (OECD) looked at 12 countries (Australia, Belgium, Canada, Cyprus, Czech Republic, Finland, France, Ireland, Japan, Poland, the UK, and the US) and the factors that helped or hindered APN role development [35]. Similar to this study, the OECD found the following were affecting APN development: support and opposition from the medical and nursing community, issues related to regulation, and limitations of educational opportunities. It is noteworthy that the same practice climate issues still surround advanced practice a decade later.

## Limitations

This study had several limitations, primarily the ability to reach and receive results from as wide a range of countries as hoped. For the most part the study relied on convenience sampling of ICN or ICN NP/APNN affiliated countries. The country-specific firewall challenges resulted in changing the study from an online survey to a word document submission as well as extending the deadline. Of the countries responding, several had very low response rates (fewer than three respondents), especially for a study relying on evidence of occurrence for reporting purposes. Another limitation was that the survey was only available in English, thereby limiting respondents not fluent in English or limiting understanding of some nursing terms. Similarly, the language surrounding credentialing and certification caused confusion, which made analysis cumbersome and required extensive cross referencing. Finally, one country included (Ecuador) did not have credentialed APNs outside of those credentialed by US agencies, which limited the applicability of the survey for nurses from that country.

## Implications for Practice and Policy

While considerable progress has been made for advanced practice nursing, significant challenges persist. The global community seems to be awakening to the strong possibility that APNs may be part of the solution for access to healthcare services, especially in the context of universal health coverage (UHC) [36,37,38]. UHC means all individuals receive needed health services without suffering economic hardship and is one of the primary goals the United Nations agreed on when they created and adopted 17 Sustainable Development Goals (SDGs), aimed at solving many environmental, economic, and political problems around the world [39]. Indeed, because good health is fundamental to education and economic security, many of the SDGs would be impacted by UHC. For this reason, policy makers, educators, and clinicians need to consider how and to what degree the APN can mitigate some of the challenges around UHC. Suggested strategies might include the following:

1. Educate stakeholders on the importance of titling, title protection, and role consistency. Seek out opportunities to crosswalk titles and role wherever feasible and in a progressive stepwise fashion. Create a consistency of process and language so that APN research about the profession is productive, meaningful, and transferrable.
2. Perform similar work as recommendation #1 for consistencies and efficiencies of education. Agreement on a core advanced practice curriculum would be helpful and would lead to accreditation models that might function on a broader scale, allowing flexibility and mobility.
3. Explore and educate stakeholders about regulatory models and their critical importance in shaping the foundations of sound regulations that protect the public and the provider community.
4. Educate APNs about all aspects of health policy and why they need to influence or become policy makers. Every APN needs to understand they are an ambassador to their own future—by providing high-quality health care, effectively and efficiently, they win lifelong support from patients and communities and that communicates well to administrators and regulators.
5. Promote the importance of APN data keeping and data analysis to the profession, administrators, and regulators. This includes personal data keeping by practicing APNs, as well as data keeping by organizations, jurisdictions, and countries who employ APNs. One data set that would be particularly useful to initiate and obtain at intervals would be a consistent data set of APN role development around the world. Only through the keeping of a consistent data set will advances and gaps in progress be documented.
6. Examine funding mechanisms that support the education, regulatory, and practice systems that equip APNs for the level of care they can provide when supported. This includes funding for systems of accreditation, credentialing, curriculum and program development, reimbursement systems, and others.
7. Raise public awareness of APN care. This includes everything from communication via media campaigns to more personal communications aimed at colleagues, medical staff, administrative bodies, insurance companies, reimbursement platforms, and ministries of health.

## Conclusion

The recommended strategies are ambitious but fundamental to the process of creating systems where APNs can develop and thrive. APNs can serve their patients and communities in complex and patient-centered ways when systems of education and healthcare delivery are thoughtfully designed. While country- and culture-specific issues continue to exist, this study identified common policy and practice issues critical to the APN role which need consideration to optimize the care and leadership these nurses offer patients, healthcare systems, and countries. Indeed, if the world is sincerely working toward universal healthcare coverage, APNs should be a meaningful part of the solution.

## Additonal File

The additonal file for this article can be found as follows:

10.5334/aogh.3698.s1 Appendices. Appendix A and B.

## References

[1] International Council of Nurses. Guidelines on advanced practice nursing 2020. Geneva; 2020. https://www.icn.ch/system/files/documents/2020-04/ICN_APN%20Report_EN_WEB.pdf.

[2] Levine N. What does a nurse practitioner do? [Internet]. New York: Cedars-Sinai; 2019 Nov 9. https://www.cedars-sinai.org/blog/nurse-practitioners.html#:~:text=Broadly%20speaking%2C%20NPs%20are%20trained,even%20from%20hospital%20to%20hospital.

[3] Canadian Nurses Association. Advanced practice nursing: A Pan-Canadian framework [Internet]. Ottawa; 2019. https://cna-aiic.ca/-/media/cna/page-content/pdf-en/advanced-practice-nursing-framework-en.pdf?la=en&hash=76A98ADEE62E655E158026DEB45326C8C9528B1B.

[4] Schober M. Loretta Ford lecture. Lecture presented at Fellows of the American Association of Nurse Practitioners 2021 Virtual Winter Meeting; 2021 Jan 9, United States.

[5] World Health Organization. Global health workforce shortage to reach 12.9 million in coming decades [Internet: press release]. Geneva; 2013 Nov 13. https://apps.who.int/mediacentre/news/releases/2013/health-workforce-shortage/en/index.html.

[6] World Health Organization. Classifying health workers: Mapping occupations to the international standard of classification. Geneva; 2010 April 29. https://docplayer.net/12682455-Classifying-health-workers-mapping-occupations-to-the-international-standard-classification.html.

[7] International Labor Office. International standard classification of occupations. Geneva; 2012. https://www.ilo.org/wcmsp5/groups/public/@dgreports/@dcomm/@publ/documents/publication/wcms_172572.pdf.

[8] Research Subgroup of the International Council of Nurses Nurse Practitioner/Advanced Practice Nursing Network. Update: International survey of nurse practitioner/advanced practice nursing roles [Internet]. Geneva; 2001. https://international.aanp.org/Practice/Survey2001.

[9] Roodbol P. Survey results presented for the ICN International Practitioner/Advanced Practice Nursing Conference; 2004 Gronigen, Netherlands.

[10] Pulcini J, Jelic M, Gul R, Loke AY. An international survey on advanced practice nursing education, practice and regulation. J Nurs Scholarsh. 2010 Mar; 43(1): 31–39. DOI: 10.1111/j.1547-5069.2009.01322.x 20487184

[11] Heale R, Buckley R. An international perspective of advanced practice nursing regulation. Int Nurs Rev. 2015 Sept 6; 62(3): 421–429. DOI: 10.1111/inr.12193 26058446

[12] Maier C, Aiken L. Task shifting from physicians to nurses in primary care in 39 countries: A cross-country comparative study. Eur J Publ Health. 2016 Aug; 26(6): 927–934. DOI: 10.1093/eurpub/ckw098 27485719

[13] APRN Joint Dialogue Group. Consensus model for APRN regulation: Licensure, accreditation, certification & education. S.l; 2008. https://ncsbn.org/Consensus_Model_Report.pdf.

[14] Finnamore H. Celebrating nurse anesthetist education—Alice Magaw (1860–1928): Mother of anesthesia [Internet]. Rochester: Mayo Clinic; 2015 Jan 26. https://sharing.mayoclinic.org/2015/01/26/celebrating-nurse-anesthetist-education-alice-magaw-1860-1928-mother-of-anesthesia/.

[15] Lusk B, Cockerham AZ, Keeling AW. Highlights from the history of advanced practice nursing in the United States. In: Tracy MF, O’Grady ET (eds.), Hamric and Hanson’s advanced practice nursing. 6th ed. New York: Elsevier; 2019. p. 1–24.

[16] International Council of Nurses. International perspectives: ICN announces its position on advanced nursing roles. Int Nurs Rev. 2002 Dec; 49: 202. https://onlinelibrary.wiley.com/doi/epdf/10.1046/j.1466-7657.2002.00155_2.x. 12542083

[17] Larson MS. The rise of professionalism: A sociological analysis. Berkeley, CA: University of California Press; 1978.

[18] World Health Organization. Global health workforce statistics database [Internet]. Geneva. 2022. https://www.who.int/data/gho/data/themes/topics/health-workforce.

[19] Schober M. Global emergence of nurse practitioner/advanced practice nursing roles. J Am Assoc Nurse Pract. 2018 April; 30(4): 182–184. DOI: 10.1097/JXX.0000000000000029 29757785

[20] Zug K, Cassiani S, Pulcini J, Garcia A, Aguirre-Boza F, Park J. Advanced practice nursing in Latin American and the Caribbean: Regulation, education and practice. Rev Lat Am Enfermagem. 2016 Aug 8; 24: e2807. DOI: 10.1590/1518-8345.1615.2807 27508923 PMC4990050

[21] Glarcher M, Lex KM. Advanced nursing in Austria under consideration of outcome measurement. Z Evid Fortbild Qual Gesundhwes. 2020 Sept 1; 155: 11–16. DOI: 10.1016/j.zefq.2020.06.012 32811773

[22] Lopes-Júnior LC. Advanced practice nursing and the expansion of the role of nurses in primary health care in the Americas. SAGE Open Nurs. 2021 May 24; 7: 1–4. DOI: 10.1177/23779608211019491 PMC8150638 34104717

[23] Aaron EM, Andrews CS. Integration of advanced practice providers into the Israeli healthcare system. Isr J Health Policy Res. 2016 Feb 22; 5: article 7. DOI: 10.1186/s13584-016-0065-8 PMC4763450 26909141

[24] Christmals CD, Armstrong SJ. The essence, opportunities and threats to advanced practice nursing in Sub-Saharan Africa: A scoping review. Heliyon. 2019; 5(10): 1–20. DOI: 10.1016/j.heliyon.2019.e02531 PMC6812225 31667384

[25] Dlamini CP, Khumalo T, Nkwanyana N, Mathunjwa-Dlamini TR, Macera L, Nsibandze BS, et al. Developing and implementing the family nurse practitioner role in Eswatini: Implications for education, practice, and policy. Ann Glob Health. 2020; 86(1): 1–10. DOI: 10.5334/aogh.2813 32477886 PMC7243836

[26] Tsay S, Tsay S, Ke C, Chen C, Tung H. Analysis of nurse practitioner scope of practice in Taiwan using the Longest policy cycle model. J Am Assoc Nurse Pract. 2019 March; 31(3): 198–205. DOI: 10.1097/JXX.0000000000000127 30550389

[27] Kooienga SA, Carryer JB. Globalization and advancing primary health care nurse practitioner practice. J Nurse Pract. 2015 Jul–Aug; 11(8): 804–811. DOI: 10.1016/j.nurpra.2015.06.012

[28] Maier C, Aiken L. Expanding clinical roles for nurses to realign the global health workforce with population needs: A commentary. Isr J Health Policy Res. 2016 Jun; 5: 1–5. DOI: 10.1186/s13584-016-0079-2 27280014 PMC4897947

[29] Scanlon A, Bryant-Lukosius D, Lehwaldt D, Wilkinson J, Honig J. International transferability of nurse practitioner credentials in five countries. J Nurse Pract. 2019 Jul–Aug; 15(7): 487–493. DOI: 10.1016/j.nurpra.2019.02.007

[30] Blair K. Practice issues: Regulation including prescriptive authority and title protection, certification, clinical privileges/credentialing, and liability. In: Blair K, Jansen MP (eds.), Advanced practice nursing. 2018; 187–210. 6th ed. New York: Springer. DOI: 10.1891/9780826161536.0010

[31] American Nurses Association. Title “nurse” protection [Internet]. Maryland; 2020. https://www.nursingworld.org/practice-polcy/advocacy/state/title-nurse-protection/.

[32] Styles M, Affara F. ICN on regulation: Towards 21st century models. Geneva: ICN; 1997.

[33] Institute for Credentialing Excellence. ICE guide to understanding credentialing concepts [Internet]. Washington, DC; 2005. www.credentialingexcellence.org.

[34] Maier C. Nurses in advanced roles in primary care: Policy levers for implementation. OECD Proceedings; 2016, Jun 27, Paris, France: OECD. https://www.oecd.org/els/health-systems/Item-2a-Nurses-advanced-roles-Maier-University-Technology.pdf.

[35] Delamaire M, Lafortune G. Nurses in advanced roles: A description and evaluation of experiences in 12 developed countries. OECD health working paper No. 54 [Internet]. Paris: Organization for Economic Cooperation and Development; 2010. DOI: 10.1787/Skmbrcfms5g7-en

[36] World Health Organization. A vision for primary health care in the 21st century: Towards universal health coverage and the sustainable development goals [Internet]. Geneva; 2018. https://apps.who.int/iris/bitstream/handle/10665/328065/WHO-HIS-SDS-2018.15-eng.pdf?sequence=1&isAllowed=y. 10.1016/S0140-6736(18)32556-X 30343843

[37] World Bank Group. ECSA Eastern, Central and Southern African Region. Education and labor markets for nurses. Challenges and opportunities [Internet]. Washington, DC; 2021. https://www.icn.ch/sites/default/files/inline-files/WBG_ECSA%5B12%5D.pdf.

[38] World Health Organization. WHO global strategic directions for nursing and midwifery (SDNM) 2021–2025 [Internet]. Geneva; 2021. https://www.who.int/publications/m/item/global-strategic-directions-for-nursing-and-midwifery-2021-2025.

[39] World Health Organization. Universal health coverage [Internet]. Geneva; 2019. https://www.who.int/news-room/fact-sheets/detail/universal-health-coverage-(uhc).

