# Supplementary material for: Advanced Practice Nursing Roles, Regulation, Education, and Practice: A Global Study
Source: Ann Glob Health. 2022 Jun 16;88(1):42. doi: 10.5334/aogh.3698 (PMC9205376; doi:10.5334/aogh.3698)
Supplement: Appendices. — Appendix A and B. [file agh-88-1-3698-s1.pdf]

Appendices

Appendix A. Practice Role

Country	*n*	Title for NP/APN	Title Protection	Types of Positions Evident There	Professional Role Issues Evident There	Skills Evident There
**Australia**	5	Nurse Practitioner, re: midwifery- generally non-nurse undergraduate degree though registered nurses can obtain Master of Midwifery Practice	Yes	*Physician's office<br>*Independent nursing practice<br>*Hospital based clinic<br>*Community based clinic<br>*Public health or ministry of health<br>*Hospital<br>*Home health care facility<br>*Long term care facility<br>*Specialty practice<br>*Mental health<br>*School health<br>*Occupational or workplace health<br>*Faculty position<br>*Administration<br>*Research	*Maintains personal liability/indemnity/ malpractice insurance<br>*Carries their own caseload of clients/patients<br>*Has the authority to prescribe medications<br>*Has the authority to dispense/furnish medications<br>*Has the authority to order diagnostic tests<br>*Refers to other health care professionals<br>*Seeks out consultation with other health professionals<br>*Other professionals seek out consultations with the NP/APN<br>*Receives direct payments for services for clients<br>*Receives payment for serviced from other sources (i.e., National Health Service [NHS] or insurance companies)<br>*Practices independently without physician supervision<br>*Practice activities for these types of NP/APN actions varies by jurisdiction	*Skin lesion removal/skin biopsy/superficial abscess incision and drainage/foreign body removal<br>*Nail removal<br>*Cerumen removal<br>*Fluorescein stain to eye<br>*Joint aspirations and injections<br>*Therapeutic injections (such as for osteoporosis or rheumatoid arthritis)<br>*Wound management<br>*Surgical debridement<br>*Splinting<br>*Casting<br>*Suturing<br>*Pulmonary function tests and office spirometry<br>*Pap tests<br>*In-dwelling contraceptive management (IUDs) and/or long term hormonal implantation<br>*Microscopy<br>*12 lead ECG interpretation<br>*X-ray interpretation<br>*Midwifery<br>*Critical care/emergency acts (intubation, chest tube insertion/removal, central line insertion)<br>*Nerve blocks<br>*Sedation for procedures
**Botswana**	2	Nurse Practitioner, Clinical Nurse Specialist, Community Health Nurse, Midwife	Yes	*Physician's office<br>*Independent nursing practice<br>*Hospital based clinic<br>*Community based clinic<br>*Public health or ministry of health<br>*Hospital<br>*Home health care facility<br>*Long term care facility<br>*Specialty practice<br>*Mental health<br>*School health<br>*Occupational or workplace health<br>*Faculty position<br>*Administration<br>*Research	*Carries their own caseload of clients/patients<br>*Has the authority to prescribe medications,<br>*Has the authority to dispense/furnish medications<br>*Has the authority to order diagnostic tests<br>*Refers to other health care professionals<br>*Seeks out consultation with other health professionals<br>*Other professionals seek out consultations with the NP/APN<br>*Receives direct payments for services for clients<br>*Practices independently without physician supervision<br>*Practice activities for these types of NP/APN	*Skin lesion removal/skin biopsy/superficial abscess incision and drainage/foreign body removal<br>*Cerumen removal<br>*Wound management<br>*Casting<br>*Suturing<br>*Pap tests<br>*In-dwelling contraceptive management (IUDs) and/or long term hormonal implantation<br>*12 lead ECG interpretation<br>*X-ray interpretation<br>*Midwifery<br>*Critical care/emergency acts (intubation, chest tube insertion/removal, central line insertion)<br>*Sedation for procedures

					actions varies by jurisdiction	
**Canada**	85	Nurse Practitioner, Clinical Nurse Specialist	Yes	*Physician's office<br>*Independent nursing practice<br>*Hospital based clinic<br>*Community based clinic<br>*Public health or ministry of health<br>*Hospital<br>*Home health care facility<br>*Long term care facility<br>*Specialty practice<br>*Mental health<br>*School health<br>*Occupational or workplace health<br>*Faculty position<br>*Administration<br>*Research	*Maintains personal liability/indemnity/ malpractice insurance<br>*Carries their own caseload of clients/patients<br>*Has the authority to prescribe medications<br>*Has the authority to dispense/furnish medications<br>*Has the authority to order diagnostic tests<br>*Refers to other health care professionals<br>*Seeks out consultation with other health professionals<br>*Other professionals seek out consultations with the NP/APN<br>*Receives direct payments for services for clients<br>*Receives payment for serviced from other sources (i.e., National Health Service [NHS] or insurance companies)<br>*Practices independently without physician supervision<br>*Practice activities for these types of NP/APN actions varies by jurisdiction	*Skin lesion removal/skin biopsy/superficial abscess incision and drainage/foreign body removal<br>*Nail removal<br>*Cerumen removal<br>*Fluorescein stain to eye<br>*Joint aspirations and injections<br>*Therapeutic injections (such as for osteoporosis or rheumatoid arthritis)<br>*Wound management<br>*Surgical debridement<br>*Splinting<br>*Casting<br>*Suturing<br>*Pulmonary function tests and office spirometry<br>*Pap tests<br>*In-dwelling contraceptive management (IUDs) and/or long term hormonal implantation<br>*Microscopy<br>*12 lead ECG interpretation<br>*X-ray interpretation<br>*Midwifery<br>*Critical care/emergency acts (intubation, chest tube insertion/removal, central line insertion)<br>*Nerve blocks<br>*Sedation for procedures
**Chile**	3	Advanced Practice Nurse, Master of Advanced Practice Nursing	No	*Hospital based clinic<br>*Public health or ministry of health<br>*Hospital<br>*School health<br>*Faculty position<br>*Administration	*No evidence	*Wound management<br>*Midwifery
**Ecuador (role not established outside US agencies)**	1	Nurse Practitioner (where present in US agencies)	No	*Other (role present only in US agencies)	*Other (role present only in US agencies)	*Other (role present only in US agencies)
**Finland**	4	Nurse Practitioner, Clinical Nurse Specialist	No	*Physician's office<br>*Independent nursing practice<br>*Hospital based clinic<br>*Community based clinic<br>*Public health or ministry of health<br>*Hospital<br>*Home health care facility<br>*Long term care facility<br>*Specialty practice<br>*Mental health<br>*School health<br>*Occupational or workplace health<br>*Faculty position<br>*Administration<br>*Research	*Carries their own caseload of clients/patients<br>*Has the authority to prescribe medications<br>*Has the authority to dispense/furnish medications<br>*Refers to other health care professionals<br>*Seeks out consultation with other health professionals<br>*Other professionals seek out consultations with the NP/APN<br>*Receives payment for serviced from other sources (i.e., National Health Service [NHS] or insurance companies)	*Wound management<br>*Splinting<br>*Casting<br>*Suturing<br>*Pulmonary function tests and office spirometry<br>*Pap tests<br>*In-dwelling contraceptive management (IUDs) and/or long term hormonal implantation<br>*12 lead ECG interpretation<br>*Midwifery<br>*Critical care/emergency acts (intubation, chest tube insertion/removal, central line insertion)

					*Practices independently without physician supervision<br>*Practice activities for these types of NP/APN actions varies by jurisdiction	
**France**	4	Nurse in Advanced Practice (for NP and CNS)	Yes	*Physician's office<br>*Hospital based clinic<br>*Community based clinic<br>*Hospital<br>*Specialty practice<br>*Research	*Carries their own caseload of clients/patients<br>*Has the authority to prescribe medications<br>*Has the authority to dispense/furnish medications<br>*Has the authority to order diagnostic tests<br>*Refers to other health care professionals<br>*Seeks out consultation with other health professionals<br>*Other professionals seek out consultations with the NP/APN<br>*Receives direct payments for services for clients<br>*Practice activities for these types of NP/APN actions varies by jurisdiction	*Therapeutic injections (such as for osteoporosis or rheumatoid arthritis)<br>*Pulmonary function tests and office spirometry
**Germany**	3	Advanced Practice Nurse	No	*Hospital based clinic<br>*Hospital<br>*Specialty practice<br>*Faculty position<br>*Administration<br>*Research	*Maintains personal liability/indemnity/ malpractice insurance<br>*Carries their own caseload of clients/patients<br>*Seeks out consultation with other health professionals<br>*Other professionals seek out consultations with the NP/APN	*Joint aspirations and injections<br>*Wound management<br>*Casting<br>*Pulmonary function tests and office spirometry<br>*12 lead ECG interpretation<br>*X-ray interpretation<br>*Midwifery<br>*Critical care/emergency acts (intubation, chest tube insertion/removal, central line insertion)
**Ghana**	3	Nurse Practitioner	No	*Physician's office<br>*Independent nursing practice<br>*Hospital based clinic<br>*Public health or ministry of health<br>*Hospital<br>*Mental health<br>*Faculty position	*Has the authority to prescribe medications,<br>*Has the authority to order diagnostic tests<br>*Refers to other health care professionals<br>*Seeks out consultation with other health professionals<br>*Other professionals seek out consultations with the NP/APN<br>*Receives payment for serviced from other sources (i.e., National Health Service [NHS] or insurance companies)<br>*Practice activities for these types of NP/APN actions varies by jurisdiction,	*Skin lesion removal/skin biopsy/superficial abscess incision and drainage/foreign body removal<br>*Wound management<br>*Suturing<br>*X-ray interpretation<br>*Critical care/emergency acts (intubation, chest tube insertion/removal, central line insertion)
**Hungary**	1	Nurse in Advanced Practice	Yes	*Other (first graduates just finished so unclear)	*Other (first graduates just finished so unclear)	*Skin lesion removal/skin biopsy/superficial abscess incision and drainage/foreign body removal

						*Nail removal<br>*Cerumen removal<br>*Joint aspirations and injections<br>*Wound management<br>*Surgical debridement<br>*Splinting<br>*Casting<br>*Suturing<br>*Pulmonary function tests and office spirometry<br>*Pap tests<br>*12 lead ECG interpretation<br>*X-ray interpretation<br>*Critical care/emergency acts (intubation, chest tube insertion/removal, central line insertion)<br>*Nerve blocks<br>*Sedation for procedures
**Israel**	1	Expert Nurse	Yes	*Specialty practice	*No evidence	*Midwifery
**Italy**	1	No specific title	No	*Independent nursing practice<br>*Hospital based clinic<br>*Community based clinic<br>*Long term care facility<br>*Faculty position<br>*Research	*Maintains personal liability/indemnity/ malpractice insurance<br>*Refers to other health care professionals<br>*Seeks out consultation with other health professionals<br>*Other professionals seek out consultations with the NP/APN	*Skin lesion removal/skin biopsy/superficial abscess incision and drainage/foreign body removal<br>*Nail removal<br>*Cerumen removal<br>*Fluorescein stain to eye<br>*Joint aspirations and injections<br>*Wound management<br>*Surgical debridement<br>*Splinting<br>*Casting<br>*Suturing<br>*Pulmonary function tests and office spirometry<br>mmm=pap tests<br>*12 lead ECG interpretation
**Jamaica**	1	Nurse Practitioner	Yes	*Public health or ministry of health<br>*Hospital<br>*Specialty practice<br>*Mental health<br>*Faculty position	*Carries their own caseload of clients/patients<br>*Has the authority to prescribe medications<br>*Has the authority to order diagnostic tests<br>*Refers to other health care professionals<br>*Seeks out consultation with other health professionals<br>*Other professionals seek out consultations with the NP/APN	*Cerumen removal<br>*Wound management<br>*Pap tests<br>*In-dwelling contraceptive management (IUDs) and/or long term hormonal implantation<br>*X-ray interpretation<br>*Midwifery
**Kenya**	2	Nurse Specialist	No	*Specialty practice<br>*Faculty position<br>*Administration<br>*Research	*Refers to other health care professionals<br>*Seeks out consultation with other health professionals	*Pap tests<br>*In-dwelling contraceptive management (IUDs) and/or long term hormonal implantation<br>*Midwifery<br>*Sedation for procedures
**Netherlands**	39	Master in Advanced Nursing Practice, Nurse Specialist	Yes	*Physician's office<br>*Independent nursing practice<br>*Hospital based clinic<br>*Community based clinic<br>*Public health or ministry of health<br>*Hospital	*Maintains personal liability/indemnity/ malpractice insurance<br>*Carries their own caseload of clients/patients<br>*Has the authority to prescribe medications<br>*Has the authority to	*Skin lesion removal/skin biopsy/superficial abscess incision and drainage/foreign body removal<br>*Nail removal<br>*Cerumen removal<br>*Fluorescein stain to eye<br>*Joint aspirations and injections

				*Home health care facility<br>*Long term care facility<br>*Specialty practice<br>*Mental health<br>*School health<br>*Occupational or workplace health<br>*Faculty position<br>*Administration,<br>*Research<br>*Other (EMS, prehospital care)	dispense/furnish medications<br>*Has the authority to order diagnostic tests<br>*Refers to other health care professionals<br>*Seeks out consultation with other health professionals<br>*Other professionals seek out consultations with the NP/APN<br>*Receives direct payments for services for clients<br>*Receives payment for serviced from other sources (i.e., National Health Service [NHS] or insurance companies)<br>*Practices independently without physician supervision<br>*Practice activities for these types of NP/APN actions varies by jurisdiction	*Therapeutic injections (such as for osteoporosis or rheumatoid arthritis)<br>*Wound management<br>*Surgical debridement<br>*Splinting<br>*Casting<br>*Suturing<br>*Pulmonary function tests and office spirometry<br>*Pap tests<br>*In-dwelling contraceptive management (IUDs) and/or long term hormonal implantation<br>*Microscopy<br>*12 lead ECG interpretation<br>*X-ray interpretation<br>*Midwifery<br>*Critical care/emergency acts (intubation, chest tube insertion/removal, central line insertion)<br>*Nerve blocks<br>*Sedation for procedures
**New Zealand**	4	Nurse Practitioner	Yes	*Physician's office<br>*Independent nursing practice<br>*Hospital based clinic<br>*Community based clinic<br>*Public health or ministry of health<br>*Hospital<br>*Home health care facility<br>*Long term care facility<br>*Specialty practice<br>*Mental health<br>*School health<br>*Occupational or workplace health<br>*Faculty position<br>*Administration<br>*Research	*Maintains personal liability/indemnity/ malpractice insurance<br>*Carries their own caseload of clients/patients<br>*Has the authority to prescribe medications<br>*Has the authority to dispense/furnish medications<br>*Has the authority to order diagnostic tests<br>*Refers to other health care professionals<br>*Seeks out consultation with other health professionals<br>*Other professionals seek out consultations with the NP/APN<br>*Receives direct payments for services for clients<br>*Receives payment for serviced from other sources (i.e., National Health Service [NHS] or insurance companies)<br>*Practices independently without physician supervision<br>*Practice activities for these types of NP/APN actions varies by jurisdiction	*Skin lesion removal/skin biopsy/superficial abscess incision and drainage/foreign body removal<br>*Nail removal<br>*Cerumen removal<br>*Fluorescein stain to eye<br>*Joint aspirations and injections<br>*Therapeutic injections (such as for osteoporosis or rheumatoid arthritis)<br>*Wound management<br>*Surgical debridement<br>*Splinting<br>*Casting<br>*Suturing<br>*Pulmonary function tests and office spirometry<br>*Pap tests<br>*In-dwelling contraceptive management (IUDs) and/or long term hormonal implantation<br>*12 lead ECG interpretation<br>*X-ray interpretation<br>*Critical care/emergency acts (intubation, chest tube insertion/removal, central line insertion)<br>*Nerve blocks *Sedation for procedures
**Portugal**	3	Clinical Specialist, Specialist Nurse	Yes	*Physician's office<br>*Independent nursing practice<br>*Hospital based clinic<br>*Community based clinic<br>*Public health or	*Refers to other health care professionals<br>*Seeks out consultation with other health professionals<br>*Other professionals seek out consultations	*Skin lesion removal/skin biopsy/superficial abscess incision and drainage/foreign body removal<br>*Cerumen removal<br>*Wound management<br>*Splinting

				ministry of health<br>*Hospital<br>*Home health care facility<br>*Long term care facility<br>*Specialty practice<br>*Mental health<br>*School health<br>*Faculty position<br>*Administration<br>*Research	with the NP/APN<br>*Receives payment for serviced from other sources (i.e., National Health Service [NHS] or insurance companies)<br>*Practices independently without physician supervision	*Casting<br>*Suturing<br>*Pap tests<br>*X-ray interpretation<br>*Midwifery<br>*Critical care/emergency acts (intubation, chest tube insertion/removal, central line insertion)
**Republic of Ireland**	5	Advanced Nurse Practitioner, Registered Advanced Nurse Practitioner, Registered Advanced Midwife Practitioner	Yes	*Physician's office<br>*Independent nursing practice<br>*Hospital based clinic<br>*Community based clinic<br>*Public health or ministry of health<br>*Hospital<br>*Home health care facility<br>*Long term care facility<br>*Specialty practice<br>*Mental health<br>*Faculty position<br>*Administration<br>*Research	*Maintains personal liability/indemnity/ malpractice insurance<br>*Carries their own caseload of clients/patients<br>*Has the authority to prescribe medications<br>*Has the authority to dispense/furnish medications<br>*Has the authority to order diagnostic tests<br>*Refers to other health care professionals,<br>*Seeks out consultation with other health professionals<br>*Other professionals seek out consultations with the NP/APN<br>*Receives payment for serviced from other sources (i.e., National Health Service [NHS] or insurance companies)<br>*Practices independently without physician supervision	*Skin lesion removal/skin biopsy/superficial abscess incision and drainage/foreign body removal<br>*Nail removal<br>*Cerumen removal<br>*Fluorescein stain to eye<br>*Joint aspirations and injections<br>*Therapeutic injections (such as for osteoporosis or rheumatoid arthritis)<br>*Wound management<br>*Surgical debridement<br>*Splinting<br>*Casting<br>*Suturing<br>*Pulmonary function tests and office spirometry<br>*Pap tests<br>*In-dwelling contraceptive management (IUDs) and/or long term hormonal implantation<br>*12 lead ECG interpretation<br>*X-ray interpretation<br>*Midwifery<br>*Critical care/emergency acts (intubation, chest tube insertion/removal, central line insertion)<br>*Nerve blocks<br>*Sedation for procedures
**Singapore**	3	Advanced Practice Nurse	Yes	*Hospital based clinic<br>*Community based clinic<br>*Hospital<br>*Home health care facility<br>*Long term care facility<br>*Specialty practice<br>*Mental health<br>*Faculty position<br>*Administration	*Carries their own caseload of clients/patients<br>*Has the authority to prescribe medications<br>*Has the authority to dispense/furnish medications<br>*Has the authority to order diagnostic tests<br>*Refers to other health care professionals<br>*Seeks out consultation with other health professionals<br>*Other professionals seek out consultations with the NP/APN<br>*Receives direct payments for services for clients<br>*Receives payment for serviced from other sources (i.e., National Health Service [NHS]	*Skin lesion removal/skin biopsy/superficial abscess incision and drainage/foreign body removal<br>*Wound management<br>*Surgical debridement<br>*Splinting<br>*Casting<br>*Suturing<br>*Pap tests<br>*12 lead ECG interpretation<br>*In-dwelling contraceptive management (IUDs) and/or long term hormonal implantation<br>*X-ray interpretation<br>*Critical care/emergency acts (intubation, chest tube insertion/removal, central line insertion)

					or insurance companies)<br>*Practices independently without physician supervision<br>*Practice activities for these types of NP/APN actions varies by jurisdiction	
**Spain**	10	Nurse in Advanced Practice, Nurse Case Manager, Expert Nurse, Specialist Nurse	No	*Physician's office<br>*Independent nursing practice<br>*Hospital based clinic<br>*Community based clinic<br>*Public health or ministry of health<br>*Hospital<br>*Home health care facility<br>*Long term care facility<br>*Specialty practice<br>*Mental health<br>*School health<br>*Occupational or workplace health<br>*Faculty position<br>*Administration<br>*Research	*Maintains personal liability/indemnity/malpractice insurance<br>*Carries their own caseload of clients/patients, cc=has the authority to prescribe medications<br>*Has the authority to dispense/furnish medications<br>*Has the authority to order diagnostic tests<br>*Refers to other health care professionals<br>*Seeks out consultation with other health professionals<br>*Other professionals seek out consultations with the NP/APN<br>*Practices independently without physician supervision<br>*Practice activities for these types of NP/APN actions varies by jurisdiction	*Skin lesion removal/skin biopsy/superficial abscess incision and drainage/foreign body removal<br>*Nail removal<br>*Cerumen removal<br>*Fluorescein stain to eye<br>*Joint aspirations and injections<br>*Therapeutic injections (such as for osteoporosis or rheumatoid arthritis)<br>*Wound management<br>*Surgical debridement<br>*Splinting<br>*Casting<br>*Suturing<br>*Pulmonary function tests and office spirometry<br>*Pap tests<br>*In-dwelling contraceptive management (IUDs) and/or long term hormonal implantation<br>*Microscopy<br>*12 lead ECG interpretation<br>*X-ray interpretation<br>*Midwifery<br>*Critical care/emergency acts (intubation, chest tube insertion/removal, central line insertion)<br>*Nerve blocks<br>*Sedation for procedures
**Tanzania**	1	No specific title	No	*Hospital based clinic<br>*Public health or ministry of health<br>*Hospital<br>*Faculty position<br>*Administration<br>*Research	*Other professionals seek out consultations with the NP/APN	*No evidence
**United Kingdom**	41	Multiple	No	*Physician's office<br>*Independent nursing practice<br>*Hospital based clinic<br>*Community based clinic<br>*Public health or ministry of health<br>*Hospital<br>*Home health care facility<br>*Long term care facility<br>*Specialty practice<br>*Mental health<br>*School health<br>*Occupational or workplace health<br>*Faculty position<br>*Administration<br>*Research	*Maintains personal liability/indemnity/ malpractice insurance<br>*Carries their own caseload of clients/patients<br>*Has the authority to prescribe medications<br>*Has the authority to dispense/furnish medications<br>*Has the authority to order diagnostic tests<br>*Refers to other health care professionals<br>*Seeks out consultation with other health professionals<br>*Other professionals seek out consultations with the NP/APN	*Skin lesion removal/skin biopsy/superficial abscess incision and drainage/foreign body removal<br>*Nail removal<br>*Cerumen removal<br>*Fluorescein stain to eye<br>*Joint aspirations and injections<br>*Therapeutic injections (such as for osteoporosis or rheumatoid arthritis)<br>*Wound management<br>*Surgical debridement<br>*Splinting<br>*Casting<br>*Suturing<br>*Pulmonary function tests and office spirometry<br>*Pap tests<br>*In-dwelling contraceptive

				*Other (transport services)	*Receives direct payments for services for clients<br>*Receives payment for serviced from other sources (i.e., National Health Service [NHS] or insurance companies)<br>*Practices independently without physician supervision<br>*Practice activities for these types of NP/APN actions varies by jurisdiction	management (IUDs) and/or long term hormonal implantation<br>*Microscopy<br>*12 lead ECG interpretation<br>*X-ray interpretation<br>*Midwifery<br>*Critical care/emergency acts (intubation, chest tube insertion/removal, central line insertion)<br>*Nerve blocks<br>*Sedation for procedures
**United States**	103	APRN (Nurse Practitioner, Clinical Nurse Specialist, Certified Nurse Midwife, Certified Registered Nurse Anesthetist)	Yes	*Physician's office<br>*Independent nursing practice<br>*Hospital based clinic<br>*Community based clinic<br>*Public health or ministry of health<br>*Hospital<br>*Home health care facility<br>*Long term care facility<br>*Specialty practice<br>*Mental health<br>*School health<br>*Occupational or workplace health<br>*Faculty position<br>*Administration<br>*Research	*Maintains personal liability/indemnity/ malpractice insurance<br>*Carries their own caseload of clients/patients<br>*Has the authority to prescribe medications<br>*Has the authority to dispense/furnish medications<br>*Has the authority to order diagnostic tests<br>*Refers to other health care professionals<br>*Seeks out consultation with other health professionals<br>*Other professionals seek out consultations with the NP/APN<br>*Receives direct payments for services for clients<br>*Receives payment for serviced from other sources (i.e., National Health Service [NHS] or insurance companies)<br>*Practices independently without physician supervision<br>*Practice activities for these types of NP/APN actions varies by jurisdiction	*Skin lesion removal/skin biopsy/superficial abscess incision and drainage/foreign body removal<br>*Nail removal<br>*Cerumen removal<br>*Fluorescein stain to eye<br>*Joint aspirations and injections<br>*Therapeutic injections (such as for osteoporosis or rheumatoid arthritis)<br>*Wound management<br>*Surgical debridement<br>*Splinting<br>*Casting<br>*Suturing<br>*Pulmonary function tests and office spirometry<br>*Pap tests<br>*In-dwelling contraceptive management (IUDs) and/or long term hormonal implantation<br>*Microscopy<br>*12 lead ECG interpretation<br>*X-ray interpretation<br>*Midwifery<br>*Critical care/emergency acts (intubation, chest tube insertion/removal, central line insertion)<br>*Nerve blocks<br>*Sedation for procedures

Low response countries in shaded gray

Appendix B. Practice Climate

Country	*n*	Factors That Facilitated NP/APN Development	Who Advocated	Who Opposed	Level of NP/APN Policy Making	Level of NP/APN Organization Development
**Australia**	5	*Need for health care providers for rural or underserved areas<br>*Strong support for nursing practice<br>*Consumer demand for increased access to health care	*Government<br>*Nursing organizations within country<br>*International organization<br>*Individual nurses<br>*Individual physicians<br>*Consumers	*Physician organizations within country<br>*Individual nurses<br>*Individual physicians	*Local<br>*National	*Local<br>*National

**Botswana**	2	*Need for health care providers for rural or underserved areas<br>*Strong support for nursing practice<br>*Consumer demand for increased access to health care<br>*Shortage of physicians	*Government<br>*Nursing organizations within country<br>*Individual nurses<br>*Consumers	*No evidence	*Local<br>*National	*Local<br>*National
**Canada**	85	*Need for health care providers for rural or underserved areas<br>*Strong support for nursing practice<br>*Consumer demand for increased access to health care<br>*Shortage of physicians (one respondent cited physician shortage was in neonatal ICUs initially)	*Government<br>*Nursing organizations within country<br>*Physician organizations within country<br>*Nongovernmental/nonprofit institution within country<br>*Private institution within country<br>*International organization<br>*Individual nurses<br>*Individual physicians<br>*Consumers	*Government<br>*Physician organizations within country<br>*Nongovernmental/ nonprofit institution within country<br>*Private institution within country<br>*Individual nurses<br>*Individual physicians<br>*Consumers<br>*Insurance companies<br>*Other (pharmacists)	*Local<br>*National	*Local<br>*National
**Chile**	3	*Need for health care providers for rural or underserved areas<br>*Strong support for nursing practice<br>*Consumer demand for increased access to health care	*Nursing organizations within country<br>*Private institution within country<br>*University	*Physician organizations within country<br>*Other (unawareness of role)	*No evidence	*National
**Ecuador (role not established outside US agencies)**	1	No evidence	*Other (one university interested)	*Government<br>*Nursing organizations within country<br>*Physician organizations within country<br>*Nongovernmental/ nonprofit institution within country<br>*Individual nurses<br>*Individual physicians<br>*Insurance companies	No evidence	No evidence
**Finland**	4	*Need for health care providers for rural or underserved areas<br>*Strong support for nursing practice<br>*Consumer demand for increased access to health care	*Government<br>*Nursing organizations within country<br>*Nongovernmental/ nonprofit institution within country<br>*International organization<br>*Individual nurses<br>*Individual physicians<br>*Other (educators, researchers)	*Physician organizations within country<br>*Individual nurses<br>*Individual physicians	*Local<br>*National	*Local<br>*National
**France**	4	*Need for health care providers for rural or underserved areas<br>*Strong support for	*Government<br>*Nursing organizations within country<br>*Physician	*Government<br>*Nursing organizations within country<br>*Physician	No evidence	*Local

		nursing practice<br>*Consumer demand for increased access to health care	organizations within country<br>*Individual nurses<br>*Individual physicians	organizations within country<br>*Individual nurses<br>*Individual physicians<br>*Insurance companies		
**Germany**	3	*Need for health care providers for rural or underserved areas<br>*Strong support for nursing practice	*Government<br>*Nursing organizations within country<br>*Physician organizations within country<br>*Other (specifically nursing administration in hospitals)	*Government<br>*Nursing organizations within country<br>*Physician organizations within country<br>*Nongovernmental/ nonprofit institution within country<br>*Individual nurses<br>*Individual physicians<br>*Insurance companies<br>*Other (physician organizations favored PAs)	*Local<br>*National	*Local<br>*National
**Ghana**	3	*Need for health care providers for rural or underserved areas<br>*Strong support for nursing practice<br>*Consumer demand for increased access to health care	*Government<br>*Nursing organizations within country<br>*Individual nurses<br>*Consumers<br>*Other (one university)	*Physician organizations within country<br>*Individual physicians	*Local<br>*National	*Local
**Hungary**	1	*Need for health care providers for rural or underserved areas<br>*Strong support for nursing practice<br>*Consumer demand for increased access to health care	*University	*Physician organizations within country	*Local<br>*National	*No evidence
**Israel**	1	*No response	*No response	*No response	*No response	*No response
**Italy**	1	*Strong support for nursing practice	*International organization	*Government	*National	*Local<br>*National
**Jamaica**	1	*Need for health care providers for rural or underserved areas<br>*Consumer demand for increased access to health care	*Government<br>*Nursing organizations within country<br>*International organization.	*Physician organizations within country	*Local	*Local<br>*National
**Kenya**	2	*Other (to train more nurses in universities)	*Nursing organizations within country<br>*Individual nurses	No evidence	*Local<br>*National	*Local<br>*National
**Netherlands**	39	*Need for health care providers for rural or underserved areas<br>*Strong support for nursing practice<br>*Consumer demand for increased access to health care<br>*Shortage of physicians (one respondent cited physician shortage was in psychiatry)<br>*Respondent cited	*Government<br>*Nursing organizations within country<br>*Physician organizations within country<br>*Nongovernmental/nonprofit institution within country<br>*Private institution within country<br>*International organization<br>*Individual nurses	*Government<br>*Nursing organizations within country<br>*Physician organizations within country<br>*Nongovernmental/nonprofit institution within country<br>*Individual nurses<br>*Individual physicians<br>*Consumers<br>*Insurance companies<br>*Other	*Local<br>*National	*Local<br>*National

		dialogue about providing right (high quality) care in the most (cost) efficient way, as well as patient needs moving from ‘illness and cure’ to ‘health and behavior’	*Individual physicians<br>*Insurance companies<br>*University	(psychiatry/psychology)		
**New Zealand**	4	*Need for health care providers for rural or underserved areas<br>*Strong support for nursing practice<br>*Consumer demand for increased access to health care	*Government<br>*Nursing organizations within country<br>*International organization<br>*Individual nurses<br>*Individual physicians	*Physician organizations within country<br>*Individual nurses<br>*Individual physicians	*Local<br>*National	*Local<br>*National
**Portugal**	3	*Consumer demand for increased access to health care<br>*University	*Nursing organizations within country<br>*Individual nurses<br>*Other (PhD researchers)	*Government<br>*Physician organization within your country<br>*Individual physicians	*No evidence	*Local<br>*National
**Republic of Ireland**	5	*Need for health care providers for rural or underserved areas<br>*Strong support for nursing practice<br>*Consumer demand for increased access to health care<br>*Other (One respondent cited commission to study nursing and developed pathway for APNs)	*Government<br>*Nursing organizations within country<br>*Physician organizations within country<br>*Nongovernmental/ nonprofit institution within country<br>*Individual nurses<br>*Individual physicians<br>*Consumers	*Individual nurses<br>*Individual physicians	*Local<br>*National	*Local<br>*National
**Singapore**	3	*Need for health care providers for rural or underserved areas<br>*Strong support for nursing practice<br>*Consumer demand for increased access to health care	*Government<br>*Nursing organizations within country<br>*Physician organization within your country<br>*International organization<br>*Individual nurses<br>*Individual physicians<br>*Consumers	*Individual nurses<br>*Individual physicians	*Local<br>*National	*Local<br>*National
**Spain**	10	*Strong support for nursing practice<br>*Consumer demand for increased access to health care	*Nursing organizations within country<br>*Nongovernmental/ nonprofit institution within country<br>*Individual nurses<br>*Individual physicians<br>*Consumers	*Government<br>*Nursing organizations within country<br>*Physician organizations within country<br>*Private institution within country<br>*Individual nurses<br>*Individual physicians<br>*Insurance companies	*Local<br>*National	*Local<br>*National
**Tanzania**	1	*Strong support for nursing practice	*Government<br>*Nursing organizations within country<br>*Individual nurses	*No evidence	*Local<br>*National	*No evidence

**United Kingdom**	41	*Need for health care providers for rural or underserved areas<br>*Strong support for nursing practice<br>*Consumer demand for increased access to health care<br>*Other (reduced working hours of residents/junior doctors due to policy change)	*Government<br>*Nursing organizations within country<br>*Physician organizations within country<br>*Nongovernmental/nonprofit institution within country<br>*Private institution within country<br>*International organization<br>*Individual nurses<br>*Individual physicians<br>*Consumers<br>*Insurance companies<br>*Other (university)	*Government<br>*Nursing organizations within country<br>*Physician organizations within country<br>*Private institution within country<br>*Individual nurses<br>*Individual physicians<br>*Consumers	*Local<br>*National	*Local<br>*National
**United States**	103	*Need for health care providers for rural or underserved areas<br>*Strong support for nursing practice<br>*Consumer demand for increased access to health care<br>*Other (reduced working hours of residents/junior doctors due to policy change)	*Government<br>*Nursing organizations within country<br>*Physician organizations within country<br>*Nongovernmental/nonprofit institution within country<br>*Private institution within country<br>*International organization<br>*Individual nurses<br>*Individual physicians<br>*Consumers<br>*Insurance companies<br>*Other (university)	*Government<br>*Nursing organizations within country<br>*Physician organizations within country<br>*Nongovernmental/nonprofit institution within country<br>*Private institution within country<br>*International organization<br>*Individual nurses<br>*Individual physicians<br>*Consumers<br>*Insurance companies<br>*Other (media)	*Local<br>*National	*Local<br>*National

Low response countries in shaded gray
